# Research Progress on Immune Evasion of *Mycoplasma hyopneumoniae*

**DOI:** 10.3390/microorganisms12071439

**Published:** 2024-07-16

**Authors:** Bin Jiang, Ying Zhang, Gaojian Li, Yanping Quan, Jianhong Shu, Huapeng Feng, Yulong He

**Affiliations:** Department of Biopharmacy, College of Life Sciences and Medicine, Zhejiang Sci-Tech University, Hangzhou 310018, China; 202220901029@mails.zstu.edu.cn (B.J.); 2023210901106@mails.zstu.edu.cn (Y.Z.); ligaojian@126.com (G.L.); quanyanping@zstu.edu.cn (Y.Q.); shujianhong@zstu.edu.cn (J.S.); fenghuapeng@zstu.edu.cn (H.F.)

**Keywords:** *Mycoplasma hyopneumoniae*, swine, immune evasion, immune system

## Abstract

As the main pathogen associated with enzootic pneumonia (EP), *Mycoplasma hyopneumoniae* (Mhp) is globally prevalent and inflicts huge financial losses on the worldwide swine industry each year. However, the pathogenicity of Mhp has not been fully explained to date. Mhp invasion usually leads to long-term chronic infection and persistent lung colonization, suggesting that Mhp has developed effective immune evasion strategies. In this review, we offer more detailed information than was previously available about its immune evasion mechanisms through a systematic summary of the extant findings. Genetic mutation and post-translational protein processing confer Mhp the ability to alter its surface antigens. With the help of adhesins, Mhp can achieve cell invasion. And Mhp can modulate the host immune system through the induction of inflammation, incomplete autophagy, apoptosis, and the suppression of immune cell or immune effector activity. Furthermore, we offer the latest views on how we may treat Mhp infections and develop novel vaccines.

## 1. Introduction

The main pathogen associated with endemic pneumonia (EP) in pigs, *Mycoplasma hyopneumoniae* (Mhp), is described as a highly contagious, chronically non-fatal pathogen that can affect pigs of all ages [1,2] and has a massive financial impact on the global swine industry [3]. Mhp has a worldwide geographical distribution, but the literature lacks concrete data on the prevalence of Mhp by country, as it does not require mandatory notification and does not restrict commercial trade in many countries [4,5]. It is reported that the average prevalence of Mhp in domestic pig herds worldwide is 30–80% [6]. This is concerning because Mhp is also the main pathogen responsible for porcine respiratory disease syndrome (PRDC), alongside two viruses that also contribute to the condition, porcine reproductive and respiratory syndrome virus (PRRSV) and porcine circovirus type 2 (PCV2). These work in concert, whereby Mhp infection suppresses the immune system of the host, thereby increasing the risk of infection with other pathogens, which can result in more severe respiratory clinical signs [5,7].

Immune evasion is the process by which a pathogen evades recognition and attack by the immune system of the host or suppresses the host’s immune response through its structural and non-structural products [8]. Such immune evasion is common and can be achieved by many pathogens. For instance, a few fungal species that can infect humans, such as *Candida albicans*, have mechanisms for evading immune detection and phagocytosis, including impeding the normal maturation of phagocytes and inducing lysis of phagocytes [9]. Likewise, severe acute respiratory syndrome coronavirus 2 (SARS-CoV-2), which is a type of virus, can interfere with key signaling pathways in the host, leading to the reduced production of interferon [10]. Moreover, *Staphylococcus aureus*, which is a frequent colonizer of the human population [11], can adopt a variety of strategies to achieve immune evasion, such as biofilms and genetic drift [12].

As a typical *Mycoplasma*, Mhp has a small genome size which means it needs to obtain essential nutrients, such as nucleic acids, lipids, and amino acids, from its surroundings and use them for its proliferation [13]. Its ability, despite this, to persistently grow and multiply in the lungs of swine and cause long-term chronic infection suggests that Mhp can achieve immune evasion [13,14].

However, to date, the immune evasion mechanisms and potential causative elements of Mhp infection have not been fully defined. Bringing together the latest work, this review describes the interactions between Mhp and the host immune system, along with the proposed mechanisms by which Mhp immune evasion occurs. Gaining a deeper understanding of the immune evasion of Mhp will help establish a foundation for the treatment and prevention of Mhp infections in the future.

## 2. *Mycoplasma hyopneumoniae* Achieves Immune Evasion by Altering its Structure or Function

**Figure 1:**
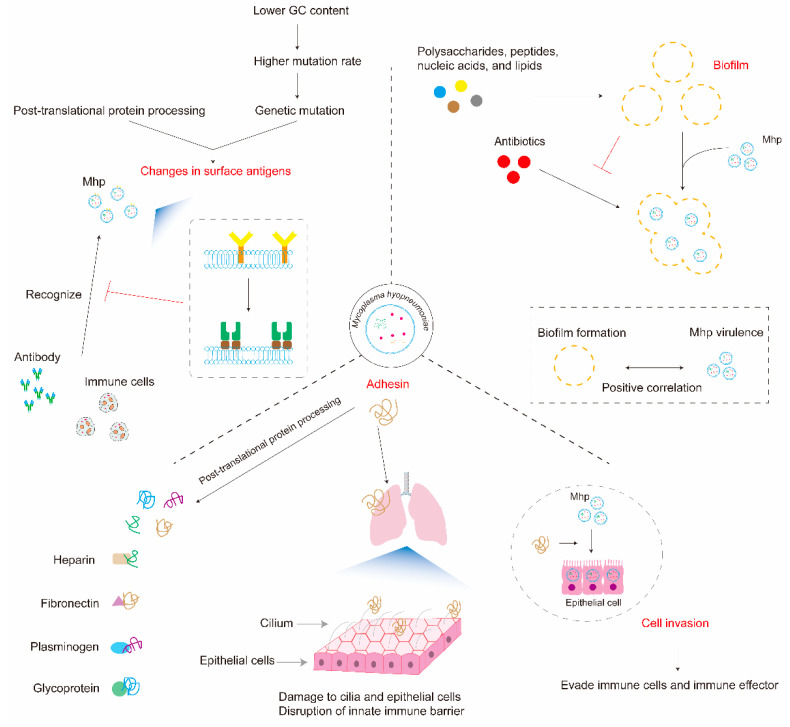
*Mycoplasma hyopneumoniae* achieves immune evasion by altering its structure or function. Mhp can mutate surface antigens via genetic mutation and post-translational protein processing. Biofilms can help Mhp resist killing by antibiotics, and the ability of Mhp to form biofilms is positively correlated with its virulence. Cellular invasion by Mhp can help it evade recognition by the immune system, and adhesins are key molecules in the invasion process. Adhesins can damage epithelial cells and cilia, disrupting the host’s intrinsic immune barrier, with protein hydrolytic cleavage resulting in diversification of the adhesin species and their functions.

Through genetic mutation and post-translational protein processing, Mhp alters the structure of surface antigens and leads to the functional diversification of some proteins, such as adhesins [13]. Beyond this, Mhp can also form biofilms [15], which have been reported to help some pathogens resist being killed by the immune system [16]. Accordingly, in this part of the article, the mechanisms of immune evasion by Mhp will be elaborated in this regard (Figure 1).

### 2.1. Genetic Mutation and Post-Translational Protein Processing

*Mycoplasmas* can cause persistent infections in the host [17], since many *Mycoplasma* genes undergo frequent and random mutations [18,19]. The expression, size, and antigenic structure of proteins on the surface of a *Mycoplasma* are altered when the gene is mutated, ultimately leading to numerous functionally and morphologically diverse phenotypes of *Mycoplasmas*, providing a mechanism for them to evade the host immune response.

Niu et al. [20] showed that GC-poor regions have a higher mutation rate than GC-rich regions. Compared with other species, the Mhp genome has a lower GC content [13], which indicates that Mhp is more prone to genetic mutations. In support of this, studies have confirmed the presence of genetic mutations between different strains of Mhp, resulting in genomic diversity [21]. These mutations will, in turn, lead to changes in the surface antigens of Mhp, such as P36 [22]. *Mycoplasma* surface antigenic proteins are mutated in three main ways [23], namely, ON/OFF switching [18,24,25], size variation [26,27], and domain shuffling [28,29]. However, these mutative mechanisms have not yet been reported in Mhp, leaving it an open question as to how genetic mutations lead to changes in the surface antigens of Mhp, which remains to be further investigated. Complicating the picture, post-translational protein processing also then leads to structural changes in Mhp surface antigens, such as P46 [30].

Beyond surface antigens, it has been found that genetic mutations and post-translational protein processing also lead to the diversification of the types and functions of other proteins in Mhp [31], which contributes to immune evasion [32,33]. For example, P146, after post-translational protein processing, can produce P50_P146_, P40_P146_, and P85_P146_, which function as adhesins [34]. Adhesins such as P159 [35] and P116 [36] are, in turn, equally capable of post-translational protein processing. In the subsequent section, we will return to adhesins for a further discussion of their role in Mhp immune evasion.

In conclusion, on the one hand, genetic mutations and post-translational protein processing lead to changes in surface antigens that help Mhp evade recognition by the immune system; on the other hand, they also enrich the variety and function of Mhp proteins, contributing to immune evasion.

### 2.2. Biofilms

Biofilms consist of microbial cells that attach to a substratum or each other [37]. Extracellular matrix, a defining feature of biofilms, is composed of polysaccharides, proteins, extracellular DNA and other minor components, whose physical and chemical properties and specific interactions can protect cells from unfavorable environmental conditions [38].

A series of studies has shown that some *Mycoplasmas* can form biofilms [39,40,41,42], though not all *Mycoplasmas* [37]. In addition, even for the same type, there are differences in its strains’ abilities to form biofilms. For example, Chen et al. [43] showed that the capacity of *Mycoplasma gallisepticum* in chickens to form biofilms varied greatly among strains.

Two of the most important functions of biofilms are helping pathogens to resist host defenses and enhancing their stress resistance [44]. For example, biofilms can resist antibodies [16], protect cells from lytic effects of complement [45], and minimize phagocytosis and cytokine expression by macrophage cells [46].

Studies have confirmed that Mhp can resist being killed by antibiotics thanks to the functioning of the biofilms that it forms [15]. Moreover, interestingly, the high or low virulence of Mhp is positively correlated with its ability to form biofilms [47]. However, it remains to be determined whether there is an interaction between the biofilms of Mhp and the host immune system.

### 2.3. Adhesion and Invasion

Early studies qualified *Mycoplasma* as an extracellular pathogen, but subsequent studies have found it within eukaryotic cells [8]. And Raymond et al. [48] found that Mhp can reside intracellularly within porcine epithelial cells. By residing within cells, *Mycoplasma* can evade being recognized and killed by the immune system of the host, thus achieving immune evasion [8].

Adhesion appears to be a prerequisite for the pathological effects of *Mycoplasmas* [8], and adhesins are key factors in the adhesion process. Adhesins are a class of biomolecules, usually proteins or glycoproteins, on the surface of pathogens. Many pathogens can attach to host cells via adhesins, which have importance for their colonization. Mhp binds tightly to the cilia of porcine respiratory epithelial cells both in vivo and in vitro [49], and this binding is specific, i.e., Mhp binds only to receptors located on porcine respiratory epithelial cells. When it adheres, the expression of cilia-related genes will be reduced, leading to cilia damage [50]. Consequently, epithelial cells are also damaged [51], destroying the host’s intrinsic immune barrier and ultimately leading to host disease.

Molecules such as heparin can block the binding of Mhp to cilia [52], but heparin, fibronectin, and plasminogen are major sites for adhesin recognition and binding, and some adhesins can also bind to glycosaminoglycans in the host (Table 1). The binding of adhesins prevents these molecules from performing their biological functions properly, which contributes to the survival of Mhp in the host.

Most adhesins possess assorted repeat units, which can bind to different molecules, such as Mhp271 [57]. In addition, multiple repeat units are often required for adhesion, with the most typical example being the ciliary adhesin P97, which requires at least eight R1 repeat units for its binding to porcine cilia [62]. Some adhesins can also act as virulence factors for Mhp in pathogenesis, such as MHJ_0461 [63] and P116 [36].

Adhesins often undergo proteolytical processing [64], which produces different protein forms with their own adhesion properties. Adhesins are proteolytically hydrolyzed on surfaces, and so the capacity of Mhp to act in this way and cleave its secreted proteins selectively offers the pathogen an extraordinary ability to alter the structure of its surface [33], leading to diversification of the types and functions of adhesins, which helps immune evasion.

## 3. *Mycoplasma hyopneumoniae* Generates Immune Evasion by Modulating the Host Immune System

Mhp infection may achieve the regulation of the immune system, though the mechanisms for it remain largely unknown. Going forward, elucidating the complex interactions between Mhp and the immune system should help us to better understand the molecular mechanisms underlying the occurrence of Mhp immune evasion.

### 3.1. Virulence Factors

Virulence factors are a group of components, such as toxins, enzymes, and surface molecules, which enable pathogens to cause disease, and such virulence factors are instrumental in the immune evasion of Mhp [65].

Mhp can encode multiple virulence factors that may modulate or disrupt the function of the immune system, which contributes to immune evasion by Mhp [65]. The most common virulence factors are adhesins, such as P146 [66,67] and P97 [68], but some enzymes, like nicotinamide adenine dinucleotide-dependent flavin oxidoreductase [69] and NADH oxidase (NOX) [70,71] can also act as virulence factors of Mhp. In addition, the various lipoproteins encoded by Mhp, such as mhp164, mhp345, and mhp379 [65], are likewise an important class of virulence factors.

**Figure 2:**
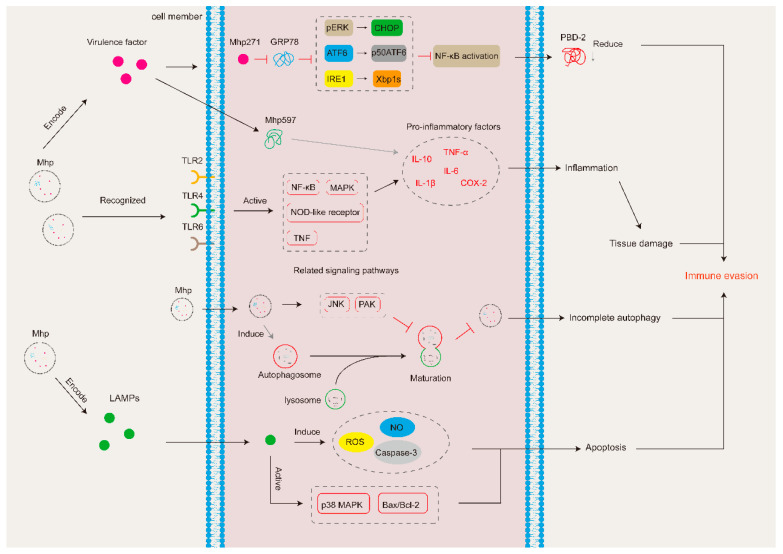
*Mycoplasma hyopneumoniae* generates immune evasion by modulating the host immune system. Mhp271 can inhibit the GRP78-related signaling pathway, resulting in reduced expression of PBD-2. When recognized by Toll-like receptors, Mhp activates signaling pathways such as MAPK, inducing excessive inflammatory cytokine expression and ultimately leading to associated organ or tissue damage. Moreover, Mhp597 can promote the expression of related inflammatory cytokines, which are involved in regulating the inflammatory response. Furthermore, Mhp induces incomplete autophagy by regulating JNK- and PAK-signaling pathways. Mhp infection leads to apoptosis, through a process that involves the expression of ROS, NO, and Caspase-3. Meanwhile, the p38 MAPK- and Bax/Bcl-2 signaling pathways are activated.

Some virulence factors can interact with the immune system to help Mhp survive in the host and eventually achieve immune evasion (Figure 2). For instance, GRP78 is a major regulator of the unfolded protein response, and Mhp271 can interact with it to reduce the production of porcine beta-defensin 2 (PBD-2), which promotes Mhp adhesion and infection [72,73]. Mhp390 (P68), meanwhile, induces the expression of multiple inflammatory factors and leads to apoptosis in normal host cells [74]. Additionally, lipoprotein P65 may play an essential role in the nutritional requirements of Mhp for long-chain fatty acids [75], which favors the survival of Mhp in the host body.

### 3.2. Inflammatory Response

After invading the organism, Mhp will mainly colonize the lungs of the host and elevate the expression of numerous inflammatory cytokines [76,77], such as COX-2, TNF-α, IFN-γ, IL-1β, IL-2, and other interleukins [74,78,79,80,81,82], which ultimately leads to a pathological inflammatory response in the host’s organs or tissues.

Although Mhp infection induces the expression of multiple inflammatory cytokines, the molecular mechanisms behind this remain poorly explained. To date, it is understood that Mhp has a molecular mechanism that affects key signaling pathways in host cells [32]. Moreover, Hwang et al. [83] demonstrated that inflammatory cytokine production is promoted by Mhp through the regulation of the NF-κB- and MAPK-signaling pathways, and similar mechanisms were also confirmed by Bai et al. [84]. In addition, after invading the organism, Mhp can regulate other signaling pathways, such as NOD-like [85] and TNF [86], though how it does so is still largely unknown. Furthermore, Mhp can use certain molecules in the host to promote inflammation. For example, Mhp infection promotes the expression of the pregnane X receptor (PRX), which induces the body to express substantial amounts of IL-6 and IL-8 [87].

Mhp infection can cause lung damage [88], and studies have confirmed that an elevated expression of inflammatory cytokines, such as IL-1β and IL-6, is associated with lung lesions during infection [89,90], which suggests that inflammatory cytokines are critical in the formation and spread of lung lesions. Moreover, lung homeostasis is essential for the normal physiological functioning of the lungs, and it has been reported that Mhp secretes two proteases (MHJ_0659 and MHJ_0522) that can degrade molecules related to the regulation of lung homeostasis [91]. Its subsequent disruption affects the performance of the lungs’ normal physiological functions, which may result in immune evasion by Mhp.

Generally, the inflammatory response is essential in the response of the immune system to pathogen infection, but, in this case, it seems that the inflammatory response induced by Mhp infection damages the host tissue cells, resulting in lung tissue lesions, which affects the normal functioning of the immune system and thus supports Mhp immune evasion (Figure 2).

### 3.3. Autophagy and Apoptosis

Autophagy can degrade pathogens that invade cells by forming autophagic lysosomes. Autophagy is induced to serve this purpose after Mhp invades the host, but it seems that the autophagosomes induced by this process fail to fuse properly with lysosomes to form autophagic lysosomes, resulting in incomplete autophagy [92]. Wen et al. [93] demonstrated that Mhp can induce such incomplete autophagy in host cells by regulating the JNK- and PAK-signaling pathways. The above studies showed that, in this way, Mhp can evade immune attack and achieve intracellular survival and proliferation (Figure 2).

Apoptosis, meanwhile, is an innate defense mechanism that can limit pathogen invasion by removing infected cells [94]. Nevertheless, after invading an organism, Mhp adopts a variety of ways to induce apoptosis in normal cells, for instance, PK15 cells [80], alveolar macrophages [95,96], peripheral blood mononuclear cells (PBMCs) [84], and lung epithelial cells [97].

Excessive production of NO and ROS and the activation of caspase-3 are key factors in apoptosis. Mhp lipid-associated membrane proteins (LAMPs) can induce alveolar macrophages to produce excessive amounts of NO and ROS and activate caspase-3, ultimately leading to apoptosis [96], with type I signal peptidases playing an important role in caspase-3-induced apoptosis [98]. Normally, alveolar macrophages recruit neutrophils, which phagocytose and remove pathogens invading the organism, restoring the lungs to their normal physiological condition [99]. However, Mhp-induced apoptosis of alveolar macrophages prevents this process from proceeding properly. In addition, LAMPs can also cause apoptosis by activating the p38 MPAK- and Bax/Bcl-2-signaling pathways [84,97].

Excessive apoptosis of immune cells will lead to immunosuppression of the organism, weakening its immune response and increasing the probability of reinfection [65], which favors the survival of Mhp in the host through immune evasion (Figure 2).

### 3.4. Inhibition of the Activity of Immune Effectors or Immune Cells

#### 3.4.1. Anti-Immunoglobulin Strategies

Humoral immunity plays an important immunoprotective role after a pathogen breaks through the host’s innate immune system. Immunoglobulins, as effectors of humoral immunity, are the most important means for the body to respond to invading pathogens. However, many *Mycoplasmas* can inactivate immunoglobulins, causing the immune system to fail to function properly [100], which ultimately results in immune evasion. *Mycoplasmas* prevent the normal function of immunoglobulins through two main actors: proteases and immunoglobulin-binding proteins (IBPs) [101]. For example, *Ureaplasma urealyticum* can secrete a protease that cleaves IgA [102,103,104], and *Mycoplasma gallisepticum* and *Mycoplasma synoviae* can express a cysteine protease CysP, which can cleave IgG into Fab and Fc [105]. IBPs, meanwhile, block effector function of immunoglobulins. For example, *Mycoplasma hominis* can produce a protein M, which has a high affinity for binding all human and non-human immunoglobulin G (IgG), thereby blocking antigen–antibody binding [106]. However, similar mechanisms have not been reported in Mhp.

There is an MIB (*Mycoplasma* Ig-binding protein)-MIP (*Mycoplasma* Ig protease) dual-protein system, where, after the MIB binds to IgG tightly, the MIP can exert its protease activity to cut off the VH structural domains of the IgG, leading to the loss of its biological activity [100]. Similar mechanisms have been found in many *Mycoplasmas* [107,108,109]. Homologous sequence comparison revealed the presence of three homologous MIB genes and three homologous MIP genes, respectively, in Mhp [100]. Considering that Mhp has homologous MIB and MIP genes, IBPs and protease activity can be inferred, but the relevant mechanism requires further study.

Here, the failure of immunoglobulins to function properly by secreting immunoglobulin-binding proteins and/or proteases represents an important method of immune evasion by *Mycoplasmas*, whereby immunoglobulin cleavage upsets the balance of immune containment, tipping that in favor of pathology [110].

#### 3.4.2. Other Strategies to Inhibit the Activity of Immune Effectors or Immune Cells

The role of the complement system is to recognize and remove foreign pathogens. However, Yu et al. [111] found that Mhp can evade being killed by the complement system by binding factor H with the help of EF-Tu. Specifically, the neutrophil extracellular trap (NET), secreted by activated neutrophils, can capture and kill extracellular pathogens, but Mhp can degrade the NET formed in vivo during infection [112]. Beyond this, Mhp can also block the normal functioning of the host immune system by inhibiting the antigen-presenting capacity of dendritic cells (DCs) [113], reducing phagocytosis by alveolar macrophages [114] and evading phagocytosis by porcine alveolar macrophages [14].

## 4. Conclusions and Future Perspectives

**Figure 3:**
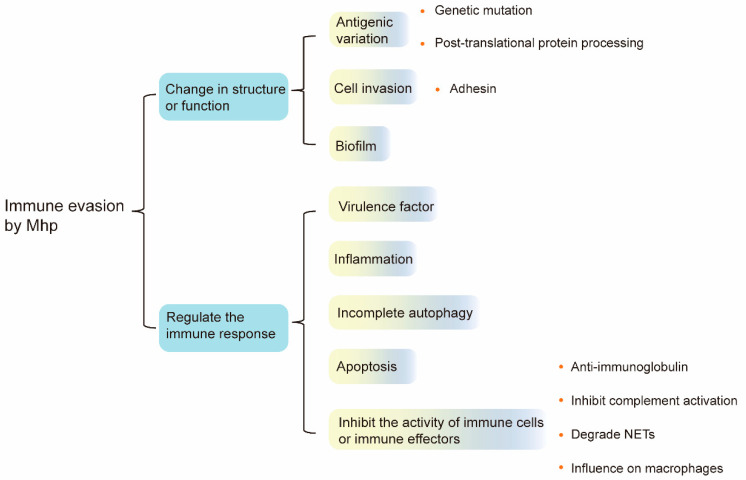
The main strategies of immune evasion by Mhp include antigenic mutation, invasion, and biofilm formation and modulation of the immune response. By inducing excessive inflammation, incomplete autophagy, apoptosis, and inhibition of immune cells or immune effector activity, Mhp can achieve the modulation of the host immune system.

The worldwide epidemic of Mhp has caused immeasurable economic losses to the swine industry, and so far, there is no particularly effective means to prevent and treat Mhp infections. The greatest challenge to such treatment is that Mhp has developed effective immune evasion strategies. Accordingly, this review summarized the mechanisms of immune evasion by Mhp (Figure 3).

Genetic mutation and post-translational protein processing confer Mhp the ability to alter its surface antigens, and the biofilm protects Mhp from being killed by antibiotics. Moreover, Mhp can achieve cell invasion with the help of adhesins, and it modulates the host immune system through the induction of inflammation, incomplete autophagy, apoptosis, and the suppression of immune cell or immune effector activity. In addition, virulence factors also play a significant role in regulating the host immune system, and their functions can be impacted by Mhp.

Cellular invasion, on the one hand, allows Mhp to exist within host cells, protected from being recognized and killed by the immune system, and, on the other hand, it disrupts the innate immune barrier and exacerbates the effects of infection. In addition, Mhp can evade the immune system by disrupting tight junctions (TJs) and the integrity of the epithelial barrier [115] and inducing cytoskeletal rearrangements in porcine respiratory tract cells [48]. However, the molecular mechanisms of immune evasion by Mhp are still poorly understood, and there is a need for in-depth exploration of the key molecules involved in certain biological processes such as apoptosis and autophagy.

In practice, in the swine industry, the use of antibiotics to control Mhp is limited due to issues such as resistance and residual risks caused by antibiotic use [116], meaning vaccination remains the most cost-effective method for controlling the disease. Currently, inactivated and attenuated vaccines are the most widely used for Mhp, but they have the disadvantages of low antibody levels and poor protective effects [117]. Meanwhile, adhesins, as important virulence factors for Mhp, have value in the development of new genetically engineered vaccines with better immunoprotection compared to conventional vaccines, such as P97 [118]. Furthermore, chimeric vaccines, consisting of multiple antigenic epitopes, also provide good immunoprotection. For example, a recombinant multi-antigen chimeric vaccine consisting of four Mhp antigens, P97R1, P46, P95, and P42, has favorable immunogenicity [119]. Today, genetic mutations and post-translational processing of proteins provide direction in the search for new adhesins, as well as help identify more candidate epitopes for the development of multi-epitope chimeric vaccines. Additionally, since Mhp can regulate the host immune system by modulating certain signaling pathways or key molecules, ultimately causing immune evasion, some critical molecules (e.g., PRX [87]) are expected to offer new targets for drug development.

In conclusion, Mhp can achieve immune evasion in several ways. Its immune evasion not only contributes to the persistence of Mhp infection but also increases the risk of host infection with other pathogens. Unfortunately, the molecular mechanisms by which immune evasion occurs in Mhp are still poorly understood. Going forward, elucidating these mechanisms holds scientific significance for improving our understanding of persistent Mhp infections and immune responses against them, as well as provides guidance for the development of anti-Mhp drugs and novel vaccines.

## Figures and Tables

**Table 1:** Recognition binding sites of *Mycoplsama hyopneumoniae* adhesins.

Adhesin	Binding Site	Reference
P146	Plasminogen	[34]
Fructose-1,6-bisphosphate aldolase (FBA)	Fibronectin	[53]
MHJ_0194 (P123)	Proteoglycans, glycoproteins, plasminogen	[32]
Mhp182 (P102)	Fibronectin	[54,55]
Mhp107	Heparin	[56]
P116	Fibronectin, plasminogen	[36]
Mhp271	Heparin, fibronectin	[57]
Mhp493 (P216)	Heparin	[58]
Mhp183 (P97)	Heparin	[52,55]
P159	Glycosaminoglycan	[59]
MHJ_0493 (P216)	Glycosaminoglycan	[60]
Mhp683	Glycosaminoglycan	[61]

## Data Availability

The data that support the findings of this study are available from the corresponding author upon reasonable request.
